# Rat 3D Printed Induction Device (RAPID-3D): A 3D-Printed Device for Uniform and Reproducible Scald Burn Induction in Rats with Histological and Microvascular Validation

**DOI:** 10.3390/biology14040378

**Published:** 2025-04-07

**Authors:** Oana-Janina Roșca, Alexandru Nistor, Călin Brandabur, Rodica Elena Heredea, Bogan Hoinoiu, Codruța Șoica

**Affiliations:** 1 Discipline of Clinical Practical Skills, Department I Nursing, Faculty of Medicine, Victor Babeș University of Medicine and Pharmacy, 300041 Timișoara, Romania; oana-janina.rosca@umft.ro (O.-J.R.); elena-rodica.heredea@umft.ro (R.E.H.); hoinoiu@umft.ro (B.H.); 2 Department of Pharmaceutical Chemistry, Faculty of Pharmacy, Victor Babeș University of Medicine and Pharmacy, 300041 Timișoara, Romania; codrutasoica@umft.ro; 3 Plastic Surgery Department, University Hospital UZ Brussel, Vrije Universiteit Brussel, 1090 Brussels, Belgium; 4 Symme3D, 300516 Timișoara, Romania

**Keywords:** scald burn model, rat burn model, rat scald, thermal burn model, experimental rat model, 3D printing, preclinical burn research

## Abstract

Background: Scald burns are common thermal injuries in clinical settings, yet existing animal models lack standardization in burn size, exposure time, and severity control. Traditional burn induction methods, such as manual immersion or heated metal contact, suffer from high variability, limited reproducibility, and are operator-dependent, reducing their translational relevance. This study presents RAPID-3D (rat printed induction device—3D), a novel 3D-printed system designed to induce uniform and reproducible scald burns in a rat model, ensuring precise exposure control and minimal variability. Methods: RAPID-3D features four burn exposure windows (10 × 20 mm each, 10 mm spacing), allowing for controlled boiling water (100 °C, 8 s) exposure while immobilizing the anesthetized rat’s dorsum. N = 10 female Wistar rats were subjected to eight controlled burns per animal. Internal unburned control areas were used in each rat for intra-animal comparison. Burn evolution was assessed using digital planimetry, histological evaluation, and real-time microvascular perfusion analysis via laser Doppler line scanning (LDLS) at 1 h, which was repeated on day 4, 9 and 21 post-burn. Results: RAPID-3D generated highly consistent burn sizes (198 ± 3.54 mm^2^) across all rats, with low inter-animal variability. Histological analysis confirmed full-thickness epidermal necrosis and deep partial-thickness dermal damage (600–900 µm depth). Microvascular Trends: Perfusion dropped immediately post-burn, remained low at day 4, and gradually increased from day 9 onward, suggesting progressive neovascularization and vascular remodeling. RAPID-3D provides a standardized, reproducible, and clinically relevant scald burn model, eliminates operator-induced variability, enhances experimental consistency, and offers strong translational relevance for burn treatment development and wound healing research.

## Simple Summary

Burn injuries are common and can cause serious skin damage, yet scientists still struggle to create reliable laboratory models to study how burns heal and how treatments work. Many current models lack precision, making it difficult to compare results across studies. To address this, we developed a new burn model called RAPID-3D, a 3D-printed device that creates uniform, reproducible scald burns in laboratory rats. Our study tested how well this device works by measuring burn size, tissue damage, skin hydration, and blood flow over time. The results showed that RAPID-3D produces consistent burns with predictable healing patterns, closely resembling real-world burn injuries. Over 21 days, we observed tissue regeneration, scar formation, and gradual skin recovery, providing a reliable timeline for studying wound healing. This research is valuable because it improves the accuracy of burn studies, allowing scientists to develop better treatments for burn victims. By ensuring standardized and repeatable experiments, RAPID-3D could help advance new therapies, wound care techniques, and drug testing.

## 1. Introduction

Burn injuries represent a major global health burden, affecting over 11 million people annually and accounting for approximately 180,000 deaths each year, predominantly in low- and middle-income countries [1]. Beyond the immediate threat to life, burns lead to long-term complications including hypertrophic scarring, contractures, and chronic inflammation, significantly impairing patient quality of life [2]. Among thermal burns, scald injuries—caused by exposure to hot liquids or steam—are the most prevalent, particularly in pediatric and elderly populations [3,4]. In a global cohort, utilizing the World Health Organization’s Global Burn Registry, children aged 1–5 years comprised 62% of the pediatric cohort [5], with 80% of these injuries resulting from scald burns, underscoring the need for effective prevention and treatment strategies. A pediatric burns study reported that scald burns constituted 83.4% of cases, with 70% attributable to hot liquids related to cooking, such as coffee, tea, or soup [6].

Unlike contact burns, which result in immediate and well-demarcated tissue damage, scald burns induce progressive tissue injury due to a continued heat transfer and prolonged dermal exposure [7]. This dynamic injury process presents a unique challenge in burn pathophysiology and treatment development, necessitating clinically relevant and reproducible preclinical models.

Animal models play a crucial role in advancing molecular and cellular discoveries, particularly when testing new therapeutic approaches without risking patient safety [8]. Rodent models are widely used for studying burn injuries, particularly for contact and scald burns, due to their short life cycle, reproducibility, and translational potential. Existing animal models suffer from inconsistencies in burn severity and poor reproducibility due to manual induction techniques [2]. Table 1 highlights current burn induction models used in rodent studies.

While both contact and scald models in rodents are valuable for studying the local and systemic effects of thermal burns, scald burns tend to produce more widespread damages and inflammatory responses than contact burns. Table 2 compares existing contact and scald burn models in rats.

Contact burns in rodents are typically inflicted by placing a heated metal surface against the skin for a controlled duration and temperature, allowing for consistent burn depth and severity. This model is often employed to study burn progression, wound healing, and scar formation, with variations depending on the exposure time and pressure applied. The burn severity in such rodent models is controlled by adjusting the contact temperature (ranging from 54 °C to 330 °C) and the exposure duration (4 s to 5 min). Contact burns typically create localized, well-demarcated wounds and may not fully mimic the systemic effects of severe burns, such as those seen in scald or flame burns

Scald burns are induced by immersing a specific body part (usually the back or limb) in hot water or exposing the skin to hot steam, mimicking common real-world burn scenarios such as household accidents. The Walker–Mason scald model is a widely used standardized method in rats, allowing for partial- or full-thickness large-area burns that facilitate investigations into systemic inflammatory responses, infection susceptibility, and therapeutic interventions.

The most commonly used scald burn models rely on manual immersion techniques, where an animal’s skin is submerged in hot water for a predefined period. Burn severity is controlled by adjusting the water temperature (typically 60–100 °C) and exposure duration (3–30 s). Large total body surface area (TBSA) burns (10–40% TBSA) can be induced this way. While the Walker–Mason scald model and its derivative techniques are widely used, they all suffer significant drawbacks. They are operator-dependent, producing highly variable, fluctuating burn severity due to variations in immersion depth, water turbulence, and inconsistent heat transfer. This creates a significant limitation for researchers aiming to produce reliable, easily reproducible, and standardized scald burns in rats.

A recent Delphi consensus on an ideal burn model highlights the need for standardized induction methods and reproducibility to improve the translational value [1]. The ideal model proposed through the Delphi consensus should ensure a consistent burn size and depth, mimic human burn pathophysiology, eliminate variability in operator technique, and integrate a microvascular and inflammatory response assessment.

To address these challenges, we have developed the rat printed induction device—3D (RAPID-3D), a novel 3D-printed device designed to induce uniform, reproducible scald burns in rats, with a well-established and quantifiable TBSA% on the dorsal surface of the animal. This device standardizes burn size and depth, minimizes operator-dependent variability, and closely mimics the pathophysiological characteristics of human scald injuries, simultaneously reducing the number of experimental animals required to evaluate the effects of various formulations on scald healing. By providing a consistent and reliable model, RAPID-3D aims to enhance the translational relevance of preclinical burn research and facilitate the development of effective treatments.

**Figure 1:**
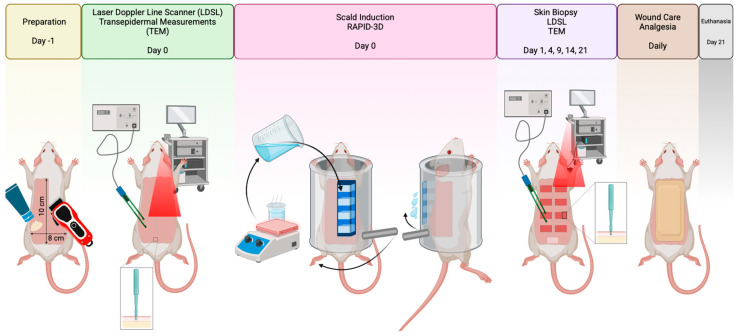
Experimental workflow for rat printed induction device—3D (RAPID-3D) scald burn induction and assessment. (Day-1): Animals underwent preparation, including dorsal hair removal and area marking (10 cm × 8 cm). (Day 0): Baseline laser Doppler line scanner (LDSL) perfusion imaging and transepidermal measurements (TEM) were performed before burn induction. Scald burns were induced using the RAPID-3D device, ensuring precise temperature and exposure time control. (Days 1, 4, 9, 14, 21): wound progression was monitored via LDSL imaging, TEM analysis, and skin biopsies for histological assessment. Daily wound care and analgesia were provided throughout the study period. (Day 21): Animals were euthanized for final tissue analysis.

In this study, we present the design and validation of RAPID-3D (Figure 1), demonstrating its efficacy in producing consistent scald injuries using histological and microvascular validation through macroscopic assessment, transdermal water loss, skin color, moisture content, a sebometer, skin pH, as well as the depth of lesion, necrosis, vascularization, epithelization, and skin perfusion imaging using Moor laser Doppler line scanner (LDLS).

## 2. Materials and Methods

### 2.1. Device Conceptualization and 3D Modeling

The central design of the RAPID-3D scald burn induction device is a hollow hexahedron with four slots in the base that provide contact between the hot water and the rat’s skin. This device can be applied to the left and right dorsal hemithorax area of the rat, resulting in 8 uniformly sized burns. Through sequential iterations of the 3D model, a working prototype of the RAPID-3D scald burn induction device was developed using SolidWorks 2024 (Dassault Systèmes, Vélizy-Villacoublay, France) software, based on our original four-slotted hollow hexahedron with additional layers of functionality and safety.

### 2.2. Study Design

Ten female Wistar rats weighing 250–300 g (average 278 g), acquired from the animal lab of the Iuliu Hatieganu University of Medicine and Pharmacy Cluj-Napoca, were used for this self-control scald induction study. All experimental protocols were carried out as per the ARRIVE guidelines [9] after receiving approval from the Research Ethics Committee of Victor Babes University of Medicine and Pharmacy, Timisoara (Nr. 17/11.03.2022). Animals were housed individually in well-ventilated cages, with highly absorbent bedding changed daily, and the cages were initially sanitized to prevent infections. They were acclimatized for 1 week before use. The animals were fed water and solid rodent chow ad libitum. Experiments were conducted under controlled conditions (room temperature maintained at 22 ± 2 °C, humidity at 50 ± 10%, and a 12 h light/dark cycle) in the animal lab of the “Pius Brânzeu” Center for Laparoscopy and Microsurgery, Timișoara.

### 2.3. Anesthesia and Animal Preparation

Before burn induction, the rats underwent a 12 h fast with free access to water.

Light sedation with isoflurane 2% was performed with the rat positioned on a heating pad. Anesthesia was performed with an intraperitoneal injection of a ketamine (100 mg/mL)/xylazine (20 mg/mL) mixture, dosed as 90 mg/kg ketamine and 10 mg/kg xylazine. The effectiveness of the anesthesia was checked by pinching one of the limbs. The hair on the animal’s back was trimmed in an area of 10 cm by 8 cm, starting from below the neck and down to the iliac bone and extending laterally until the anterior costal margins. Depilatory cream (Veet, Reckitt GMbH, Heidelberg, Germany) was applied to the trimmed area to remove any residual hairs, followed by disinfection with a povidone-iodine solution and cleaning with sterile water. During the entire experiment, the animals were provided with free access to water and solid rat chow (ad libitum). Strict aseptic procedures were followed to prevent infection transmission. The animals were routinely monitored for any signs of distress. Euthanasia was performed 21 days after scald wounds induction under full anesthesia, using 0.3 mL/kg T61 (MSD Animal Health, Intervet International B.V., Boxmeer, The Netherlands). Experimental animals were disposed of as medical waste.

### 2.4. Induction of Burns

Under general anesthesia, the experimental animal is placed in the RAPID-3D device. Depending on the animal’s weight and diameter, an appropriately sized tray (C) is selected to ensure the tightest fit possible for the animal in the cylinder (B) without restricting its breathing. Additional support may be provided in the form of yellow kitchen sponges inserted between the rat’s spine and the cylinder wall. The rat is positioned in ventrolateral decubitus, exposing the dorsal region and one hemithorax area, which have been shaved beforehand. The cuboid (A) is inserted into the cylinder slot and gently pressed against the rat’s stretched shaved skin while ensuring not to restrict its breathing amplitude. The cuboid (A) is equipped with 3 mm skirts around each of the four slots, which ensures that no hot water will spill from the margins of the slots. Once the cuboid is securely positioned against the rat’s skin, two elastic bands are fitted over the cylinder and the upper ridges of the cuboid, which is designed with notches for this purpose. The splash guards (G) are inserted into the undertray (F).

A scald burn can be induced once the rat has been securely and tightly fitted inside the cylinder. Considering the inner volume of the cuboid is 50 mL, an appropriate amount of water is prepared in advance and heated up to the desired temperature of 100 °C using an electric heating plate/magnetic stirrer (Thermoline Scientific Laboratory, Wetherill Park, Australia). Thermocouple probes ensured real-time temperature monitoring before application. A total of 40 mL of boiling water is then poured into the cuboid (A) and a timer is started by an assistant. After 8 s, the cylinder (B) is rotated swiftly using the action handle (D) to discharge the hot water into the undertray while the splash guards ensure no spillage occurs. Wearing proper safety gear is mandatory in the lab when manipulating hot liquids. After all the water is discharged from the cuboid (A), the rat is removed and inspected for water leakage in the adjacent skin. After one side of the back and hemithorax of the rat has been treated, the other side is positioned and the experiment can be repeated using the same steps. Unburned control areas within each animal are designated in the distal part of the healthy shaved skin for intra-animal comparison.

### 2.5. Aftercare of Experimental Animals

All rats were continuously monitored for distress, abnormal behavior, or signs of excessive pain. Since the experiment’s pain level is below USDA class D, analgesia was achieved with butomidor 10 mg/mL administered subcutaneously at 0.01–0.05 mg/kg every 12 h.

After the scald induction, a volumizing solution of lactated Ringer’s solution is administered intraperitoneally. The Parkland formula was used to prevent hypovolemic shock, which is as follows:

$$4\;mL\times weight\left(kg\right)\times TBSA=mL\;crystal\;loidliquid/first\;24\;h$$

A modified Elizabethan collar was applied immediately after scald induction and was kept in place for the duration of the entire experiment to prevent scratching and self-induced infection of the scald wounds.

### 2.6. Morphological Analysis of Scald Burns

The burn wounds were measured and photographed every 24 h, looking for any morphological differences in wound size, wound necrosis, or infection. Standardized digital planimetry images were taken on days 0, 4, 9, 14, and 21 after scald induction (day 0) and used to measure the burn area consistency. Burn morphology was assessed at 24 h post-burn using standardized burn severity scoring criteria. The wound size was calculated by the formula horizontal length times vertical length.

### 2.7. Transepidermal Changes of Scald Burns

The biophysical parameters of the skin were examined in vivo pre-burn and on days 0, 4, 9, 14, and 21 after scald induction using a non-invasive technique. A multi-probe adapter (MPA) (Courage-Khazaka Electronic GmbH, Cologne, Germany) was used with the following probes: Tewameter TM300 (TEWL), Mexameter MX18, and Corneometer CM825. Five determinations were made for each parameter and the average of the five measurements was noted. The temperature and humidity in the laboratory were kept constant in the range of 25 ± 1 °C and 45–50%, respectively.

### 2.8. Laser Doppler Line Scanner

The Moor laser Doppler line scanner (Moor LDLS2, Moor Instruments Inc., Wilmington, DE, USA) measures skin perfusion by quantifying blood flow proportional to the red blood cells’ average speed and their numerical concentration. This is expressed in perfusion units (PUs) and calculated using the first moment of the power spectral density, ranging from 0 to 500 PUs with 3% accuracy. The device comprises an infrared laser beam (785 nm) that performs sweeping scans of the superficial tissues, generating a 256 × 256 pixel resolution, with a 4 ms/pixel velocity. A pre-burn baseline was determined for each experimental animal, followed by scans on days 0, 4, 9, 14, and 21 after scald induction. Relative perfusion loss (%) was calculated and compared to unburned control areas.

### 2.9. Histology of Scald Burns

After sedation with isoflurane 2%, punch biopsies were harvested from the healthy control area on day 0 and from the burn areas on days 1, 4, 9, 14, and 21. Places biopsies were taken from included the epidermis, dermis, and hypodermis up to the muscle fascia, using a 2 mm diameter core biopsy (ZetMedical^®^, Arad, Romania). Bioptic fragments were fixed in 10% buffered formalin, processed in increasing concentrations of alcohol, and blocked in paraffin, which were then cut into 5 mm pieces and stained with hematoxylin and eosin (HE). Photomicrographs of tissue sections were taken at 10×, 20×, and 40× magnification with an upright bright field microscope (Olympus BX 53, Tokyo, Japan).

### 2.10. Statistical Analysis

A power analysis was performed to determine the minimum sample size required to detect the primary endpoint—burn size consistency—with sufficient statistical power. Specifically, with an anticipated coefficient of variation (CV) of less than 5%, an alpha (α) of 0.05, and power (1 − β) of 0.80, a sample size of N = 10 rats (each rat receiving eight standardized burns) was determined to be adequate to statistically validate the reproducibility and uniformity of the burn areas induced by the RAPID-3D device. The burn size consistency was assessed by calculating the mean area, standard deviation (SD), and CV across all burn locations. Additionally, the intraclass correlation coefficient (ICC) was calculated to further quantify reproducibility. Statistical analysis was performed using GraphPad Prism version 10 for Mac (GraphPad Software, Boston, MA, USA), with *p* < 0.05 considered significant.

## 3. Results

### 3.1. RAPID-3D Model

We developed the RAPID-3D prototype using SolidWorks 2024 (Dassault Systèmes) software, based on our original four-slotted hollow hexahedron. This cuboid measures 76 × 26 × 33 mm (L × W × H) and has an inner capacity of 50 mL. The cuboid’s top is open, allowing water to be poured inside. The bottom is fenestrated, presenting four equally spaced openings of 10 × 20 mm (L × W), which are placed 10 mm apart. The underside of the cuboid is fitted with 3 mm long skirts around the perimeter of each opening, which allows the cuboid to be pressed into the rat’s skin as these seal each opening and prevent any hot water spilling onto the surrounding skin. With this design, hot water could be poured inside, but rapid evacuation could not be achieved without spillage onto the adjacent rat skin or jeopardizing the operator’s safety. Therefore, an enclosure was designed to contain the experimental animal and ensure a rapid, safe evacuation of the contained hot water from the cuboid.

All parts were designed to be printable on commercially available hobby-level 3D printers with a standard printing capacity of 256 × 256 × 256 mm^3^.

**Figure 2:**
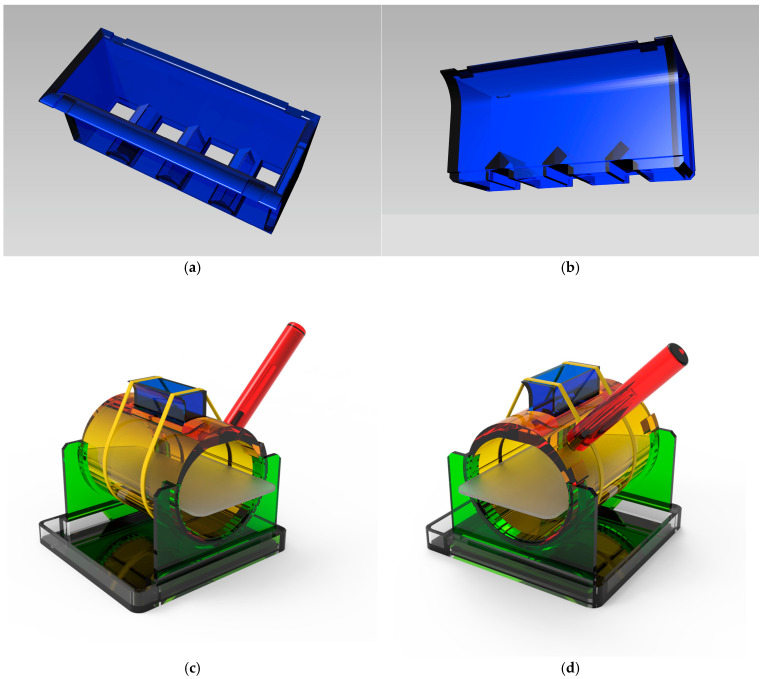
A 3D rendering of the RAPID-3D device and its components. (**a**) The cuboid with four slotted bottom openings of 10 × 20 mm, spaced 10 mm apart; perspective view. (**b**) The 3 mm-long skirts around the four openings provide a tight fit in the rat skin; bottom view. (**c**) Assembled device; perspective SE view. (**d**) Assembled device; perspective SW view.

Figure 2 depicts the resulting final model, consisting of the following:

A. A cuboid with four slotted bottom openings of 10 × 20 mm, spaced 10 mm apart and with 48 mL total capacity. The cuboid has an open top with a slanted edge and an evacuation overhang lip, and there are notches on the top edges for restraining and tensioning using two elastic bands.
B. A cylinder with a slot for the cuboid and guide rails for trays that support an anesthetized rodent (rat or mouse) of variable weight/diameter and length. Stoppers on both ends of the cylinder allow a defined arc of rotation.
C. Inner trays of variable sizes and lengths, running on the cylinder guides placed mm apart.
D. An actioning handle attached with M5 screw to the cylinder.
E. Two U-shaped supports in which the cylinder is mounted.
F. An undertray in which the two U-shaped supports are fixed, and which acts as a hot water collection tray.
G. Splash guards for the undertray, preventing spillage when emptying the cuboid.

The supplementary digital content Video S1 showcases a 3D rendering of the RAPID-3D model and the way it is actioned.

The RAPID-3D stereolithography files (.stl) required for the 3D printing of the components have been uploaded onto the protocols.io platform and can be accessed under a CC BY-NC-ND 4.0 license using the following link: https://dx.doi.org/10.17504/protocols.io.36wgqdxykvk5/v1 (accessed on 28 February 2025)

A Symme3D delta style printer was used to print out all parts. An overview of printing time, filament usage, and cost is given in Table 3. We recommend printing part A (4-slotted cuboid) and B (cylinder) in PETG, in order to withstand the thermal shock of pouring boiling water into or onto the parts. PETG also provides smoother prints, making slotting part A into part B easier. The remaining parts can be printed in PLA.

### 3.2. Burn Area Uniformity

To evaluate the reproducibility and precision of the RAPID-3D device, burn area measurements were analyzed after burn induction across all eight burn locations. The mean burn area was 198.33 mm^2^ with a standard deviation (SD) of 3.54 mm^2^, resulting in a coefficient of variation (CV) of 1.78%, indicating minimal variability in burn size across different rats.

**Figure 3:**
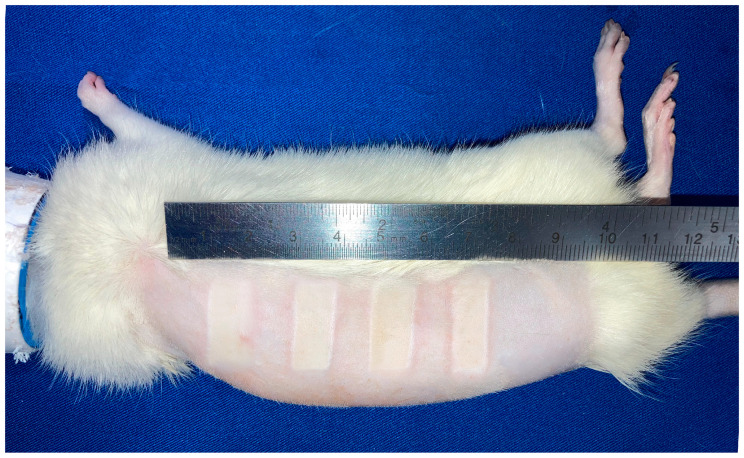
Scald wounds induced in an experimental rat using the RAPID-3D device. Four lesions were induced on the right dorsal hemithorax and dorsal lumbar region, measuring 20 × 10 mm and spaced 10 mm apart, with minimal variability in shape and size.

Further statistical analysis revealed an intraclass correlation coefficient (ICC) of −0.82, suggesting a high consistency in burn dimensions. No significant differences were observed between burn areas across animals, reinforcing the device’s reproducibility (Figure 3).

### 3.3. Wound Area Reduction over Time

**Figure 4:**
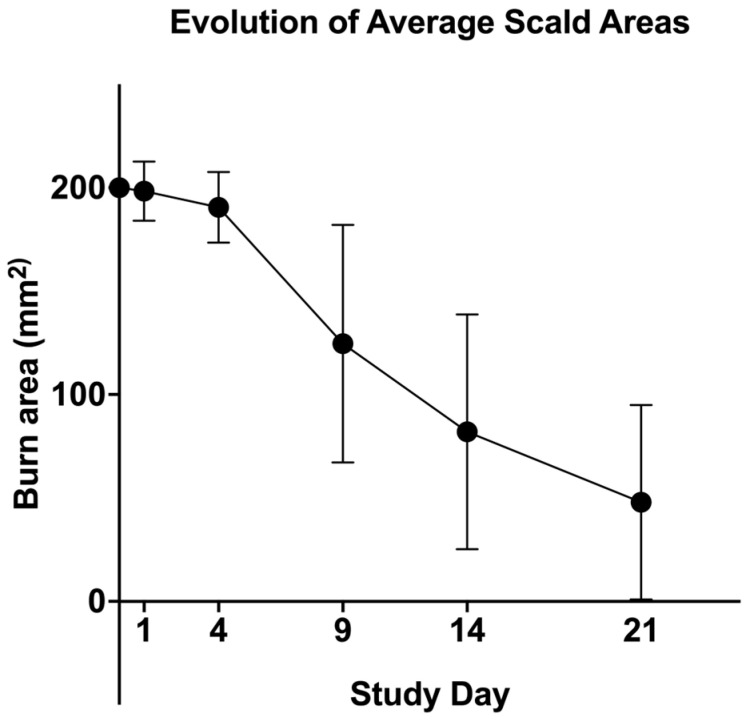
Evolution of average scald areas. The burn wound surface area was measured using standardized digital planimetry. A progressive decrease in wound size was observed over time, reflecting granulation tissue formation, re-epithelialization, and matrix remodeling.

To evaluate the progression of burn wound healing, standardized digital planimetry was used to measure wound surface area at days 0, 4, 9, 14, and 21 post-burn. Immediately after burn induction (day 0), all wounds displayed a uniform surface area, confirming the consistency of burn application. By day 4, a moderate reduction in wound size was observed, suggesting the onset of early inflammatory and proliferative phase activity. A significant contraction was noted by day 9, coinciding with granulation tissue formation and epithelial migration. Between days 14 and 21, wound closure accelerated, with a substantial reduction in surface area, indicating progressive re-epithelialization and extracellular matrix remodeling. Figure 4 displays the evolution of the average scald areas.

### 3.4. Burn Depth and Histological Validation

**Figure 5:**
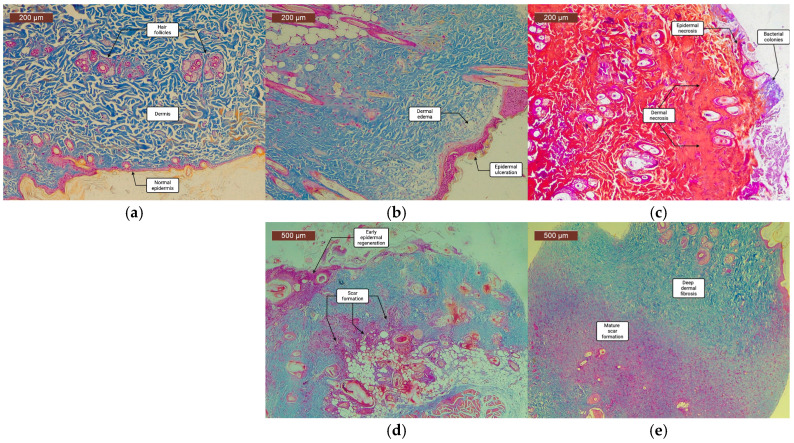
Histological progression of scald wound healing over 21 days. (Masson’s trichrome staining). (**a**) Control skin (Day 0): normal epidermis, dermis, hair follicles, and sebaceous glands. (**b**) Day 4: after scald induction: epidermal ulceration with marked dermal edema and necrosis. (**c**) Day 9: widespread epidermal and dermal necrosis, with bacterial colonies present on the wound surface. (**d**) Day 14: early epidermal regeneration, with scar formation in deep dermis and subcutaneous tissue. (**e**) Day 21: partial epidermal and superficial dermal regeneration, with persistent deep dermal fibrosis, indicating mature scar formation. Scale bars: 200 µm (**a**–**c**), 500 µm (**d**,**e**).

Histological analysis confirmed that burns induced using RAPID-3D produced deep partial-thickness injuries with a consistent depth across all burn sites. Hematoxylin and eosin (H&E) staining from biopsies taken on days 4, 9, 14, and 21 revealed a well-defined zone of coagulative necrosis extending into the reticular dermis, with a mean burn depth ranging between 600 and 900 µm. Given the reduced thickness of the rat’s skin in comparison to human skin, this corresponds well to the clinically relevant deep- and partial-thickness burns observed in humans [10]. Masson’s trichrome staining demonstrated progressive collagen degradation. Granulation tissue formation, fibrosis, inflammation, and epithelialization were assessed based on histology (Figure 5).

**Figure 6:**
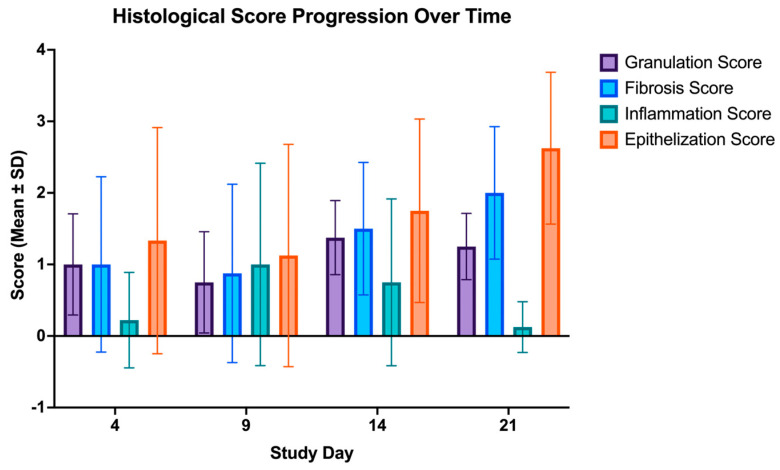
Histological score progression over time in scald wounds. Histological analysis was performed at days 4, 9, 14, and 21 post-burn to evaluate granulation tissue formation, fibrosis, inflammation, and epithelialization. Data are presented as mean ± SD. Granulation tissue peaked at day 9 and inflammation at day 14, followed by a progressive increase in fibrosis from day 14 onward, indicating tissue remodeling. Epithelialization was most evident on day 21, reflecting wound closure and epidermal regeneration.

Control skin (day 0) exhibited a normal epidermis, with hair follicles and sebaceous glands embedded in a well-organized dermal extracellular matrix. By day 4, burn wounds displayed ulceration of the epidermis, with marked dermal edema and necrosis in the upper dermis, indicating severe early tissue injury. On day 9, extensive epidermal and dermal necrosis persisted, with bacterial colonies observed on the wound surface, suggesting a transient microbial presence typical of open burn wounds. By day 14, re-epithelialization had been initiated, leading to early epidermal regeneration, while scar formation became evident in the deep dermis and subcutaneous tissue signifying active fibroblast proliferation and extracellular matrix deposition. On day 21, the epidermis and superficial dermis were partially restored, although deep dermal fibrosis persisted indicating the formation of a scar undergoing healing. Figure 6 plots the average histological score progression over time in all scald wounds.

### 3.5. Transepidermal Changes in Scald Wounds

**Figure 7:**
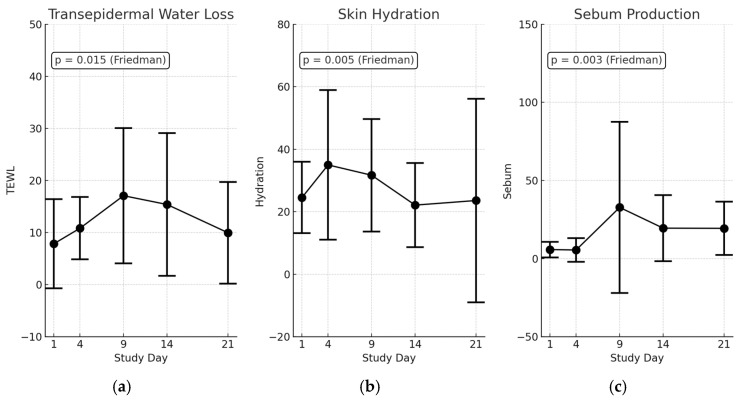
Barrier function parameters measured over the study period following scald burn induction using the RAPID-3D device. (**a**) Transepidermal water loss (TEWL), a significant increase in TEWL indicates compromised skin barrier integrity after burn injury, peaking between study days 4 and 14 (Friedman test, *p* = 0.015). (**b**) Skin hydration levels, showing a significant reduction by day 14, reflecting impaired epidermal hydration and delayed barrier recovery (Friedman test, *p* = 0.005). (**c**) Sebum production exhibited substantial variability across time points, indicating disrupted sebaceous gland function, though without statistically significant differences post-injury in pairwise comparisons (Friedman test, *p* = 0.003). Data presented as mean ± SD.

Transepidermal measurements were conducted using a Tewameter, Corneometer, and Sebometer to assess the barrier function integrity, skin hydration levels, and sebum production, respectively, following scald burn induction. These parameters provide critical insights into epidermal damage progression and barrier recovery over the 21-day study period (Figure 7). The repeated measures analysis using Friedman tests revealed statistically significant time-dependent changes across all assessed skin barrier parameters.

Transepidermal water loss (TEWL) measurements showed a significant increase post-burn, indicative of disrupted skin barrier integrity (Friedman test: χ^2^ = 14.07, *p* = 0.015). At baseline (day 0), TEWL was low (3.95 ± 2.96), reflecting normal skin conditions. By day 4, TEWL rose significantly (11.94 ± 10.14, adjusted *p* = 0.078), demonstrating acute epidermal barrier disruption. Peak disruption occurred between days 9 and 14 (18.78 ± 21.16, adjusted *p* = 0.078), consistent with ongoing epidermal impairment. TEWL declined by day 21 (12.16 ± 11.84) but remained elevated above baseline, signifying incomplete barrier recovery.

Skin hydration levels exhibited significant temporal changes (Friedman test: χ^2^ = 16.57, *p* = 0.005). Initially, hydration was normal at baseline (35.97 ± 16.40), it then slightly increased at day 4 (38.34 ± 30.52) possibly due to inflammation-induced fluid accumulation, and it then progressively decreased from day 9 to day 14 (25.08, adjusted *p* = 0.039). By day 21, hydration was at its lowest (18.55 ± 24.36), indicating persistent dehydration and compromised barrier recovery, emphasizing the clinical need for targeted hydration therapy post-burn.

Sebum production demonstrated significant variability across the study period (Friedman test: χ^2^ = 18.08, *p* = 0.003). Baseline sebum production was within normal ranges (34.96 ± 67.83), which dramatically decreased by day 4 (6.07 ± 9.13), likely due to direct glandular damage. By day 9, sebum levels transiently increased (43.81 ± 84.20), possibly as a compensatory healing response. However, fluctuating levels observed between days 14 and 21 (21.50 → 20.07) remained below the baseline, with post hoc analyses revealing no statistically significant differences from the baseline. These results suggest either high inter-animal variability or a delayed sebaceous gland recovery pattern post-injury.

Collectively, these findings statistically and descriptively confirm significant barrier disruption and partial recovery induced by the RAPID-3D burn model, validating its efficacy and consistency for standardized and reproducible experimental burn studies.

### 3.6. Microvascular Perfusion Trends (Moor LDLS Analysis)

**Figure 8:**
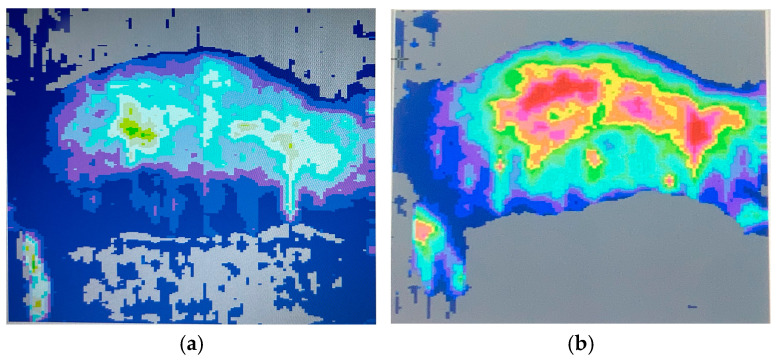
Moor LDLS images of the shaved right side of the rat. (**a**) Before scald burn induction, uniform distribution of skin perfusion is visible, with little variance. (**b**) At 14 days post scald induction, the burn lesions are over-perfused due to intense remodeling of the burn areas. A six-color palette is utilized to determine the velocity of the blood flow, with the color blue representing low velocity and the red representing high velocity.

**Figure 9:**
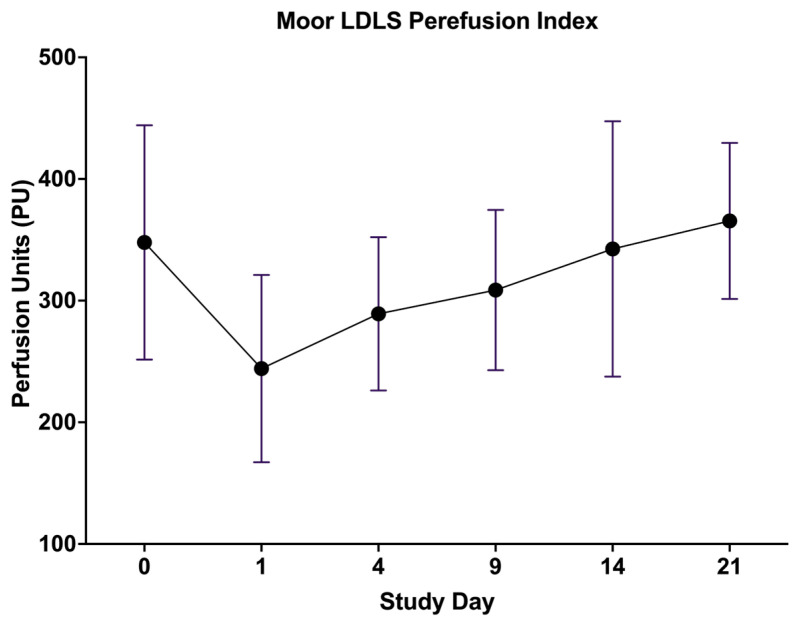
Microvascular perfusion trends in burn wounds over time. After burn induction (day 1), perfusion showed a marked decrease, indicating thermal injury-induced ischemia. Perfusion remained low on day 4, reflecting vascular stasis and microthrombosis. A gradual increase in perfusion was observed from day 9 onwards, suggesting angiogenic remodeling and capillary regeneration. By day 21, perfusion values approached and surpassed baseline levels. Data are presented as mean ± SD.

Microvascular perfusion was assessed using Moor LDLS V1 imaging software (Figure 8) at key timepoints (day 0 before the burn, day 1, 4, 9, 14, and 21 after the burn) to evaluate the ischemic response and vascular recovery post-burn. Immediately following the burn induction (day 1), a marked perfusion drop was observed (Figure 9) confirming the expected ischemic response due to acute thermal damage and microvascular stasis. Perfusion remained significantly reduced on day 4, indicating ongoing vascular impairment. By day 9, a partial recovery trend emerged, suggesting early neovascularization and capillary remodeling. The most pronounced improvement was seen between days 9 and 14, with a substantial increase in perfusion compared to earlier timepoints, reflecting progressive vascular repair. On day 21, perfusion values approached baseline levels, although residual microvascular dysfunction was still evident. These findings confirm that RAPID-3D generates a controlled ischemic injury with a predictable vascular response, closely mirroring clinical burn pathophysiology.

## 4. Discussion

### 4.1. Rationale for Developing the Device

Experimental burn models play a critical role in understanding burn pathophysiology, testing therapeutic interventions, and evaluating wound healing mechanisms. However, the lack of reproducibility in existing models has limited their translational relevance [2]. Several previously described burn models rely on heated metal probes or manual water immersion techniques, each presenting significant reproducibility challenges. Table 4 summarizes existing animal models for contact burn and scald induction and compares them to our RAPID-3D device.

### 4.2. Comparison with Other Burn Inducing Models in Rodents

Contact burn models [11,12,13] utilize heated brass, aluminum, or stainless-steel probes applied directly to the skin to induce localized full-thickness burns. These models offer burn size control but fail to replicate the progressive heat diffusion seen in real-life scald burns. Additionally, the gravitational contact method introduces variability as differences in pressure and skin adherence alter burn severity.

Scald burn models [20,21] typically employ manual immersion techniques, where rats are partially submerged in boiling water for a predefined time. While these models better mimic clinical scald burns, they suffer from uncontrolled heat diffusion, variation in immersion depth, and operator-dependent exposure times, leading to substantial inter-study variability. Additionally, large TBSA burns (>15%) can induce systemic inflammatory responses that may confound localized wound healing assessments. The contact burn severity in rodents is controlled by adjusting the contact temperature (ranging from 54 °C to 330 °C) and the exposure duration (4 s to 5 min)

Steam-based models [22] attempt to create superficial, partial-thickness, and full-thickness burns using steam exposure. However, these models face challenges in controlling the exposure duration and the risk of respiratory complications, making them less practical for standardization in small animal research.

In contrast, RAPID-3D introduces a novel, controlled method for standardized scald burn induction, addressing the variability concerns observed in both contact and immersion models.

To address these limitations, RAPID-3D was designed as an optimized burn induction system, ensuring uniform burn dimensions (20 × 10 mm per site, 10 mm apart), controlled boiling water exposure (98–100 °C for 8 s), rigid immobilization to prevent movement artifacts, precise thermal application, and rapid drainage to eliminate heat diffusion variability. No operator-induced hot water spills or safety incidents were recorded during the entire experiment. By eliminating operator-dependent inconsistencies, RAPID-3D provides a high-throughput, standardized platform for burn research, facilitating clinically relevant findings.

In comparative terms, the RAPID-3D device achieved low burn size variability (mean: 198.33 mm^2^; CV: 1.78%), significantly outperforming traditional manual immersion and contact burn models. For instance, manual immersion models typically report burn size variability ranging widely from 5 to 15%, largely attributed to inconsistent immersion depth, water turbulence, and operator-dependent exposure timing. Contact burn models, despite improved reproducibility compared to immersion, still report coefficients of variation around 5–10% due to pressure and temperature inconsistencies. Thus, RAPID-3D, with a low coefficient of variation and a uniform burn size, demonstrates methodological superiority and offers a better reproducibility

Compared to manual immersion, RAPID-3D ensures increased reproducibility by fixing exposure time and burn area. Unlike contact burns, it replicates the progressive thermal injury observed in human scald burns. Unlike flame burn models, RAPID-3D is safe, controllable, and free from flame-induced variability.

Experimental rat burn models are most often used to test various compounds or formulations developed for burn wound treatment. Older models provide only one scald area per rat, increasing the number of experimental animals needed to test multiple formulations. As such, our rationale was to create a device that can produce a high number of standardized scald burn areas in the same rat, thus drastically reducing the number of experimental rats needed. The RAPID-3D can induce eight different safe and controlled scald burns in the same rat, by applying the 4-slotted cuboid on the left and right dorsal hemithorax and dorsal lumbar area. Systemic effects were intentionally minimized by limiting TBSA to 3.8%, far below the established threshold (~10% TBSA) known to induce systemic inflammatory response syndrome (SIRS) in rodents. This methodological decision was critical for isolating local tissue responses and accurately assessing burn wound progression, epidermal regeneration, and microvascular changes without confounding systemic inflammatory effects. By focusing on localized injury, RAPID-3D allows researchers to evaluate topical treatments and local healing processes, enhancing the translational relevance.

The dorsal region of the rat was selected as the optimal site for scald burn induction due to its anatomical and behavioral advantages. Unlike other body regions, the back is difficult for the animal to access, minimizing self-inflicted trauma, licking, or scratching, which could otherwise interfere with wound progression [20]. This reduces variability in wound outcomes and ensures consistency across experimental groups. Additionally, the relatively uniform skin thickness of the rat dorsum enhances burn reproducibility, allowing for controlled depth and size across all burn sites.

Heated metal rods used to induce the burn in contact burn experiments quickly lose heat due to their low thermal inertia. Particularly problematic are metal rods heated through immersion in hot or boiling water. This method generates a highly variable contact temperature, since by the time the metal rod is taken out of the water the temperature drops by several tens of degrees. Electrically induced heat generates a more precisely controlled temperature in the metal rod, but often this cannot be controlled anymore once the rod is in contact with the skin. In contrast, the RAPID-3D allows the researcher to use a fixed amount of hot or boiling water, which has a high thermal inertia. Furthermore, using a water cooler calculator [23], the amount of time can be calculated in which a given amount of water will cool down from boiling to the desired water temperature for scald induction. Assuming an ambient temperature of 22 °C, a “cup diameter” of 1.6 cm (corresponding to the area of 200 mm^2^ for the cuboid), and a target temperature of 98 °C for the water used to pour into the cuboid, a time span of 9.5 s is needed for boiling water to reach the target temperature. Therefore, a target contact temperature of 8 s ensures that the water temperature is consistently at the desired nominal level throughout the burn induction.

Standard healing timelines for deep partial-thickness scald burns in rodents typically span between 14 and 21 days, characterized by initial inflammation and necrosis (days 1–4), granulation tissue formation and early epithelial migration (days 7–14), and progressive epithelialization and scar maturation thereafter, with near-complete epidermal regeneration usually observed around day 21. Previous studies consistently report similar healing periods, with wound contraction and re-epithelialization evident from day 9 onward [11,14,21], aligning closely with our observed timeline (necrosis resolution evident by day 9, substantial epithelialization at day 21). Thus, our findings with the RAPID-3D device reflect expected wound healing patterns in rodent burn models, further validating the clinical relevance of our experimental outcomes.

### 4.3. Limitations and Future Applications

While RAPID-3D offers significant improvements, certain limitations remain. Our experiment was conducted using a fixed temperature (98–100 °C). Future studies should evaluate lower temperatures (e.g., 70–80 °C) for partial-thickness burns, as suggested by the Delphi consensus.

No inflammatory biomarker profiling was performed due to lack of sufficient funding. Future work should include cytokine profiling (TNF-α, IL-6, VEGF) to correlate vascular damage with an immune response.

Although anesthesia and immobilization are essential for standardized burn induction, they might induce stress-related physiological responses that could influence wound healing and microvascular outcomes.

The current study focuses on early stage burn progression; further research should assess fibrosis, re-epithelialization, and collagen remodeling over extended time points (up to 28 days). While RAPID-3D minimizes operator-dependency, additional improvements could include automated water delivery and evacuation systems for high-throughput screening applications. Future studies comparing RAPID-3D directly against traditional burn models could further validate its translational value and the superiority of the RAPID-3D device.

## 5. Conclusions

The RAPID-3D system represents a significant advancement in preclinical burn modeling, offering highly standardized, reproducible, and translationally relevant scald burns. The device consistently generates uniform burn areas with minimal inter-animal variability, overcoming the limitations of traditional burn induction techniques. Histological and laser Doppler line scanning confirmed deep partial-thickness burns, which gradually progress towards wound healing, including peak granulation at day 9, fibrotic remodeling from day 14 onward, and partial epithelialization by day 21. This study establishes RAPID-3D as a robust preclinical burn model, providing a standardized platform for investigating burn pathophysiology, testing novel therapies, and advancing translational burn research.

## Figures and Tables

**Table 1:** Current burn induction models used in rodent studies.

Model	Description	Advantages	Disadvantages
Manual Immersion	Submerging a specific area of the rodent’s skin into hot water for a predetermined time.	Simple and cost-effective, widely used in various studies.	High variability due to inconsistent immersion depth and duration, operator-dependent variability, and a potential for uneven heat distribution.
Contact Burn	Applying a heated metal device directly to the skin to induce a burn.	Allows for control over burn size and depth, reproducible in terms of contact area and pressure.	May not accurately replicate the progressive nature of scald burns, a risk of deeper tissue damage due to prolonged heat exposure.
Flame Burn	Exposing the skin to an open flame for a specific duration.	Mimics certain types of thermal injuries seen in humans, useful for studying deep partial-thickness and full-thickness burns.	Difficult to control burn size and depth, safety concerns for both the animal and the researcher, and it is not representative of scald injuries.
Steam Exposure	Exposing the skin to steam to induce a burn.	Closer mimicry of scald injuries compared to dry heat methods, uniform heat distribution.	Challenges in controlling exposure parameters, and a potential for respiratory complications in animals due to inhalation of steam.
RAPID-3D Device	A 3D-printed apparatus designed to deliver controlled scald burns with precise exposure parameters.	High reproducibility and standardization, minimizes operator-dependent variability, closely mimics human scald injury characteristics, and allows for consistent burn size and depth.	Requires access to 3D printing technology, and the initial setup may be more complex compared to traditional methods.

**Table 2:** Comparative analysis of contact and scald burn models.

Feature	Contact Burn Model	Scald Burn Model
Induction Method	Heated metal surface.	Hot water immersion.
Burn Type	Localized, well-defined.	Diffuse, large area.
Depth Control	Precise with controlled pressure.	It depends on water temperature and duration.
Use in Studies	Wound healing, topical therapy, and hypertrophic scarring.	Wound healing, topical therapy, systemic effects, infection, and immune response.
Systemic Effects	Minimal.	Minimal systemic response if TBSA < 10% Strong systemic response if TBSA > 30%.
Clinical Relevance	Burns from hot objects (e.g., metals).	Scalds from hot liquids or steam.

**Table 3:** Three-dimensional model printing time, filament usage, and cost, detailed per model component.

Item	Recommended Filament	Supports Required	Printing Time (min)	Filament Usage (g)	Filament Cost (US$)
4-slotted cuboid	PETG	Yes	60	25	0.6
Cylinder	PETG	Yes	340	168	4.21
Inner trays	PLA	No	210	140	3.51
Actioning lever	PLA	No	84	16	0.41
U-shaped supports	PLA	No	100	66	1.66
Undertray	PLA	No	220	168	4.21
Splash guards	PLA	No	83	44	1.09
Total			18 h 17 min	459	15.7

**Table 4:** Comparison of previously described experimental burn models in rodents using contact or scald burns against the RAPID-3D device.

Experimental Model	Device Complexity ^1^	Burn Type	Burn Inducing Device	Device Material	Shape of Burn Lesion	Contact Area with Skin (mm)	Area of Burn Lesion (mm^2^)	Number of Lesions/Rat	Total Burn Surface Area (TBSA)	Region of Burn in Rats	Application Method	Temperature at Burn Induction	Exposure Time	Burn Degree	Experimental Animals	Gender	Weight (g)	Age	Hair Removal Method
Walker et al., 1979 [11]	++++++	Contact	Metal cylinder	Brass	Rectangular	50.8 × 279.4	14,193.5	1	42.2%	Dorsal	Rat immersion	100 °C	N/A	N/A	Rats	N/A	180–220	N/A	Shaving
Regas et al., 1992 [12]	+	Contact	Brass	Brass	Rectangular	55 × 19	1045	4	7.8%	Dorsal	Probe water immersion + Gravitational contact	N/A	20 s	Full thickness	Sprague–Dawley rats	males	300–500	N/A	Shaving
Guo et al., 2017 [13]	+++++	Contact	Aluminum bar	Aluminum	Round	20	314.2	1	3.2%	Dorsal	Gravitational contact	60–70 °C	10 s	Deep partial-thickness	Sprague–Dawley rats	males	250 ± 50	N/A	Shaving + Depilatory cream
Cai et al., 2014 [14]	++	Contact	Stainless steel rod	Stainless-steel	Round	10	78.54	3	0.5%	Dorsal	Probe water immersion + Gravitational contact	100 °C	5–20 s	Full thickness	Sprague–Dawley rats	N/A	350–400	6–8 weeks	Shaving
Venter et al., 2015 [15]	++++++	Contact	Aluminum bar	Aluminum	Round	19; 23; 32	283.53; 415.5; 804.2	1	0.7%; 1%; 2%	Central thoracodorsal	Gravitational contact + Skin fixation with syringe needles	60 °C, 70 °C, 80 °C	10 s	1. Superficial second-degree burn. 2. Deep second-degree burn. 3. Third degree-burn.	Wistar rats	males	275–300	N/A	Shaving + Depilatory cream
Andrade et al., 2017 [16]	+++	Contact	Aluminum plate	Aluminum	Round	30	706.9	1	2%	Central thoracodorsal	Gravitational contact	150 °C	5–15 s	Full thickness burn	Wistar rats	males	200–250	N/A	Shaving + Depilatory cream
Shukla et al., 2020 [17]	+++	Contact	Aluminum probe	Aluminum	Round	10	78.54	N/A	0.9%	Dorsal	Gravitational contact	60 °C, 80 °C, 100 °C	40 s	1. Superficial thickness. 2. Partial thickness. 3. Full thickness burn	Mice	females	25–30	10–12 weeks	Shaving
Hu et al., 2020 [18]	+++	Contact	Brass comb	Brass	Rectangular	10 × 20	200	8	5%	Dorsal	Probe water immersion + Gravitational contact	100 °C	8 s	Partial thickness	Sprague–Dawley rats	N/A	180–220	6–8 weeks	Shaving
Korompai et al., 2002 [19]	++++	Scald	Plastic	Polyethylene	Oval	N/A	Width of animal; axial length of planned burn	1	15–25%	1. Ventral; 2. Chest wall 3. Flanks	Gravitational contact	96 °C	10–15 s	1. Deep partial thickness burn. 2. Full thickness burns	Sprague–Dawley rats	males	85–142	4.2–5 weeks	Shaving
Abdullahi et al., 2014 [20]	++	Scald	Plastic template	Plastic	Rectangular	N/A	N/A	1	30%	Dorsal thorax, ventral lumbar	Probe immersion	100 °C	8 s	Full-thickness burn	Mice	N/A	N/A	6–8 weeks	Shaving
Davenport et al., 2019 [21]	+++	Scald	Cradle	PVC	Rectangular	50 × 136	6800	2	30.4%	Both lateral dorsal side	Rat immersion	96 °C	8 s	Full thickness burn	Sprague–Dawley rats	males	320–340	adult	Shaving + Depilatory cream
Porumb et al., 2017 [22]	++++	Scald	Steam application	Steam	Round	N/A	1256.6	1	3%	Dorsal thorax	Rat immersion	94 °C	1–7 s	1. Superficial burn wound. 2. Partial thickness. 3. Full thickness	Wistar rats	males	300 ± 20	N/A	Shaving + Depilatory cream
RAPID-3D	+++	Scald	3D printed cuboid	PETG/PLA	Rectangular	20 × 10	1600	8	3.8%	Dorsal thorax, dorsal lumbar	Controlled contact with water	98–100 °C	8 s	Full-thickness burn	Wistar rats	females	278 ± 23	6 months	Shaving + Depilatory cream

^1.^ Device complexity is rated on a continuous six-point scale, with six being the most complex. One point (+); six points (++++++). N/A—not applicable.

## Data Availability

Stereolithography files (.stl) for the RAPID-3D device have been uploaded on the protocols.io platform and can be accessed under a CC BY-NC-ND 4.0 license. The original contributions presented in this study are included in the article/Supplementary Materials. Further inquiries can be directed at the corresponding author(s).

## Supplementary Materials

The following supporting information can be downloaded at: https://www.mdpi.com/article/10.3390/biology14040378/s1, Video S1: 3D rendering of the RAPID-3D model and how it is actioned.

## Abbreviations

The following abbreviations are used in this manuscript:

RAPID-3D	Rat Printed Induction Device—3D
TBSA	Total Body Surface Area
LDLS	Laser Doppler Line Scanner
PLA	Polylactic acid
PETG	Polyethylene terephthalate glycol
SD	standard deviation
CV	coefficient of variation
ICC	intraclass correlation coefficient
SIRS	systemic inflammatory response syndrome
TEWL	Transepidermal Water Loss
